# Crystal structure of 10-ethyl-7-(9-ethyl-9*H*-carbazol-3-yl)-10*H*-pheno­thia­zine-3-carbaldehyde

**DOI:** 10.1107/S2056989017005540

**Published:** 2017-04-21

**Authors:** Vairavan Mahalakshmi, Siddan Gouthaman, Madurai Sugunalakshmi, Srinivasan Bargavi, Srinivasakannan Lakshmi

**Affiliations:** a Department of Physics, S.D.N.B. Vaishnav College for Women, Chromepet, Chennai 600 044, India; b Industrial Chemistry Polymer Division, CSIR Central Leather Research Institute, Chennai 600 020, India

**Keywords:** crystal structure, pheno­thia­zine, carbazole, carbaldehyde, C—H⋯π inter­actions, π–π inter­actions

## Abstract

The title compound, C_29_H_24_N_2_OS, contains a pheno­thia­zine moiety linked to a planar carbazole unit (r.m.s. deviation = 0.029 Å) by a C—C single bond. The pheno­thia­zine moiety possesses a typical non-planar butterfly structure with a fold angle of 27.36 (9)° between the two benzene rings. The dihedral angle between the mean planes of the carbazole and pheno­thia­zine units is 27.28 (5)°. In the crystal, mol­ecules stack in pairs along the *c*-axis direction, linked by offset π–π inter­actions [inter­centroid distance = 3.797 (1) Å]. There are C—H⋯π inter­actions present linking these dimers to form a three-dimensional structure.

## Abstract 2

In the title compound, a pheno­thia­zine moiety is linked to a planar carbazole unit (r.m.s. deviation = 0.029 Å) by a C—C single bond. Their mean planes are inclined to one another by 27.28 (5)°, and the pheno­thia­zine moiety possesses a typical butterfly structure with a fold angle of 27.36 (9)° between the two benzene rings.

## Chemical context   

Pheno­thia­zine, related to the thia­zine class of heterocyclic compounds, is very important as it occurs in various anti­psychotic drugs. Pheno­thia­zine derivatives have been used in dye-sensitized solar cells to study the effect of conjugated linkers on device performance (Kim *et al.*, 2011 ▸; Hagfeldt *et al.*, 2010 ▸). One pheno­thia­zine derivative (MCDP) is used to measure the activity of mono­amine oxidase in blood (Fujii *et al.*, 1993 ▸). They are also used as neuroleptics, sedatives, analgesics, anti-emetics and anti­histamines (Harris & Klein, 1987 ▸). Triflupromazine pheno­thia­zine hydro­chloride is one of the most potent tranquilizer drug mol­ecules (Phelps & Cordes, 1974 ▸). The pheno­thia­zine derivative thi­ethyl­perazine has the properties of an anti-emetic and is widely used for the control of post-operative vomiting, in radiation therapy and vomiting associated with malignant disease (McDowell, 1970 ▸, 1978 ▸). *N*-Alkyl­amino carbazoles show significant anti-convulsant and diuretic activity (Shoeb *et al.*, 1973 ▸). One of them, rimcazole, is a well known anti-pyretic and neuroleptic agent (Ferris *et al.*, 1986 ▸). In view of this inter­est, we have synthesized the title pheno­thia­zine derivative and report herein on its crystal structure.
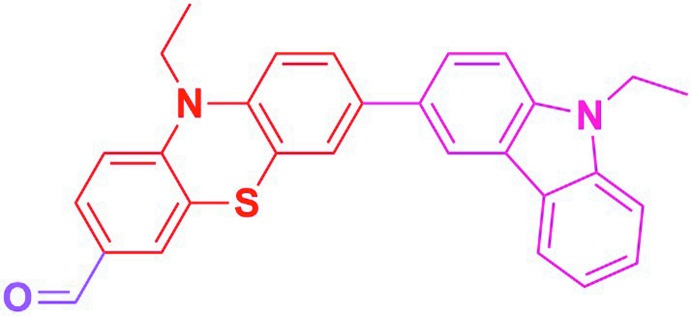


## Structural commentary   

**Figure 1:**
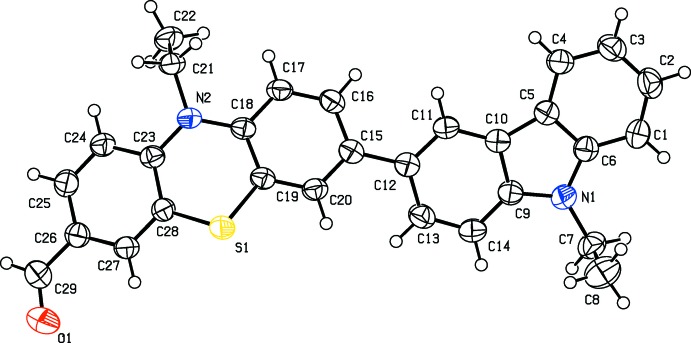
The mol­ecular structure of the title compound, with the atom labelling and displacement ellipsoids drawn at the 50% probability level.

In the title compound, the pheno­thia­zine moiety has a non-planar butterfly structure (Fig. 1 ▸), similar to that observed for 10-methyl-10*H*-pheno­thia­zine (Malikireddy *et al.*, 2016 ▸). The central six-membered ring (N2/C18/C19/S1/C28/C23) adopts a boat conformation [puckering parameters are: *Q*_T_ = 0.4567 (16) Å, θ = 102.8 (2)°, φ = 182.8 (2)°]. The fold angle of 27.36 (9)° between the two benzene rings of this moiety compares well with the values reported for similar compounds (CSD; Groom *et al.*, 2016 ▸). The dihedral angle between the planes of the two benzene rings of the carbazole ring is 2.94 (10)° and the dihedral angle between the mean planes of the carbazole and pheno­thia­zine ring systems is 27.28 (5)°. The aldehyde group is almost coplanar with the benzene ring to which it is attached, the C27—C26—C29—O1 torsion angle being 0.9 (4)°. The ethyl groups protrude out of the planes of the carbazole and pheno­thia­zine skeletons, as indicated by the torsion angles C6—N1—C7—C8 = 87.7 (3)° and C23—N2—C21—C22 = −83.2 (2)°.

## Supra­molecular features   

**Figure 2:**
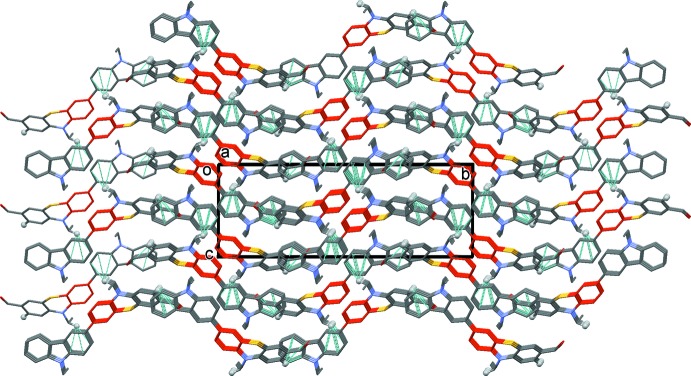
The crystal packing of the title compound, viewed along the *a* axis. The C15–C20 rings linked by π–π inter­actions are shown in red. For clarity, only the H atoms (grey balls) involved in the C—H⋯π inter­actions (dashed lines; see Table 1 ▸) have been included.

In the crystal, inversion-related mol­ecules stack in pairs along the *c*-axis direction, linked by offset π–π inter­actions [*Cg*5⋯*Cg*5^i^ = 3.7965 (11) Å, inter­planar distance = 3.5133 (8) Å, slippage = 1.439 Å, *Cg*5 is the centroid of the C15–C20 ring; symmetry code: (i) −*x* + 1, −*y*, −*z* + 2]. There are also C–H⋯π inter­actions present linking these dimers to form a three-dimensional structure (Table 1 ▸ and Fig. 2 ▸).

## Database survey   

A search of the Cambridge Structural Database (CSD, Version 5.38, update February 2017; Groom *et al.*, 2016 ▸) for compounds containing either a pheno­thia­zine, carbazole or carbaldehyde unit gave 433 hits for compounds containing a pheno­thia­zine unit, and 2293 hits for compounds containing a carbazole unit. Out of these entries, six compounds were found to possess both pheno­thia­zine and carbazole ring systems, and one compound contains all three units, pheno­thia­zine, carbazole and a carbaldehyde unit, but with the carbazole unit linked directly to the N atom of the pheno­thia­zine unit, *viz.* 10-(9-hexyl-9*H*-carbazol-3­yl)-10*H*-pheno­thia­zine-3-carbaldehyde (IWABUF; Karuppasamy *et al.*, 2017 ▸).

## Synthesis and crystallization   

To a mixture of 7-bromo-10-ethyl-10*H*-pheno­thia­zine-3-carbaldehyde (3 g, 0.0089 mol), 9-ethyl-9*H*-carbazole-3-boronic acid pinnacol ester (3.17 g, 1.1 eq.), Pd(PPh_3_)_4_ (518 mg, 5% mol) and K_2_CO_3_ (2.48 g, 2 eq.) under high vacuum was added a mixture of toluene:water (2:1). The resulting mixture was heated to reflux under an N_2_ atmos­phere for *ca* 24 h. On completion of the reaction (monitored by TLC), it was quenched by addition of saturated double-distilled H_2_O and extracted with di­chloro­methane. The organic phases were collected and washed with brine and dried over anhydrous Na_2_SO_4_ and then concentrated. The product was purified by column chromatography on silica gel using ethyl acetate:*n*-hexane (12:88, *v*/*v*) as eluent, to give the title compound as a pale-yellow crystalline solid (yield 80%). It was characterized by ^1^H NMR, ^13^C NMR, IR and ESI–MASS. Brown block-like crystals of the title compound were obtained by slow evaporation at room temperature of a solution in di­chloro­methane and aceto­nitrile (1:1 *v*/*v*).

## Refinement   

Crystal data, data collection and structure refinement details are summarized in Table 2 ▸. All H atoms were placed in calculated positions and refined using a riding-model approximation: C—H = 0.93–0.98 Å with *U*_iso_(H) = 1.5*U*_eq_(C-meth­yl) and 1.2*U*_eq_(C) for other H atoms.

## Supplementary Material

Crystal structure: contains datablock(s) I, global. DOI: 10.1107/S2056989017005540/su5357sup1.cif

Structure factors: contains datablock(s) I. DOI: 10.1107/S2056989017005540/su5357Isup2.hkl

Supporting information file. DOI: 10.1107/S2056989017005540/su5357Isup3.cml

CCDC reference: 1543611

Additional supporting information:  crystallographic information; 3D view; checkCIF report

## Figures and Tables

**Table 1:** Hydrogen-bond geometry (Å, °) *Cg*3 and *Cg*4 are the centroids of the C1–C6 and C9–C14 rings, respectively.

*D*—H⋯*A*	*D*—H	H⋯*A*	*D*⋯*A*	*D*—H⋯*A*
C21—H21*A*⋯*Cg*4^i^	0.97	2.95	3.596 (2)	125
C25—H25⋯*Cg*3^ii^	0.93	2.96	3.558 (2)	123

Symmetry codes: (i) 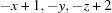; (ii) 
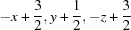
.

**Table 2:** Experimental details

Crystal data	
Chemical formula	C_29_H_24_N_2_OS
*M*_r_	448.56
Crystal system, space group	Monoclinic, *P*2_1_/*n*
Temperature (K)	296
*a*, *b*, *c* (Å)	9.4677 (6), 25.7169 (13), 9.5704 (5)
β (°)	103.681 (2)
*V* (Å^3^)	2264.1 (2)
*Z*	4
Radiation type	Mo *K*α
μ (mm^−1^)	0.17
Crystal size (mm)	0.30 × 0.25 × 0.20

Data collection	
Diffractometer	Bruker Kappa APEXII CCD
Absorption correction	Multi-scan (*SADABS*; Bruker, 2004 ▸)
*T*_min_, *T*_max_	0.677, 0.745
No. of measured, independent and observed [*I* > 2σ(*I*)] reflections	25526, 3981, 2974
*R*_int_	0.036
(sin θ/λ)_max_ (Å^−1^)	0.595

Refinement	
*R*[*F* ^2^ > 2σ(*F* ^2^)], *wR*(*F* ^2^), *S*	0.040, 0.107, 1.03
No. of reflections	3981
No. of parameters	300
H-atom treatment	H-atom parameters constrained
Δρ_max_, Δρ_min_ (e Å^−3^)	0.18, −0.20

Computer programs: *APEX2* and *SAINT* (Bruker, 2004 ▸), *SHELXS97* (Sheldrick, 2008 ▸), *SHELXL2014* (Sheldrick, 2015 ▸), *PLATON* (Spek, 2009 ▸), *Mercury* (Macrae *et al.*, 2008 ▸) and *publCIF* (Westrip, 2010 ▸).

## supplementary crystallographic information

### Crystal data

**Table:**

C_29_H_24_N_2_OS	*F*(000) = 944
*M**_r_* = 448.56	*D*_x_ = 1.316 Mg m^−^^3^
Monoclinic, *P*2_1_/*n*	Mo *K*α radiation, λ = 0.71073 Å
*a* = 9.4677 (6) Å	Cell parameters from 6254 reflections
*b* = 25.7169 (13) Å	θ = 2.4–23.8°
*c* = 9.5704 (5) Å	µ = 0.17 mm^−^^1^
β = 103.681 (2)°	*T* = 296 K
*V* = 2264.1 (2) Å^3^	Block, brown
*Z* = 4	0.30 × 0.25 × 0.20 mm

### Data collection

**Table:**

Bruker Kappa APEXII CCD diffractometer	2974 reflections with *I* > 2σ(*I*)
Bruker axs kappa axes2 CCD scans	*R*_int_ = 0.036
Absorption correction: multi-scan (SADABS; Bruker, 2004)	θ_max_ = 25.0°, θ_min_ = 2.3°
*T*_min_ = 0.677, *T*_max_ = 0.745	*h* = −11→9
25526 measured reflections	*k* = −30→30
3981 independent reflections	*l* = −11→11

### Refinement

**Table:**

Refinement on *F*^2^	Primary atom site location: structure-invariant direct methods
Least-squares matrix: full	Secondary atom site location: difference Fourier map
*R*[*F*^2^ > 2σ(*F*^2^)] = 0.040	Hydrogen site location: inferred from neighbouring sites
*wR*(*F*^2^) = 0.107	H-atom parameters constrained
*S* = 1.03	*w* = 1/[σ^2^(*F*_o_^2^) + (0.0455*P*)^2^ + 0.8313*P*] where *P* = (*F*_o_^2^ + 2*F*_c_^2^)/3
3981 reflections	(Δ/σ)_max_ = 0.004
300 parameters	Δρ_max_ = 0.18 e Å^−^^3^
0 restraints	Δρ_min_ = −0.20 e Å^−^^3^

### Special details

**Table:**

Geometry. All esds (except the esd in the dihedral angle between two l.s. planes) are estimated using the full covariance matrix. The cell esds are taken into account individually in the estimation of esds in distances, angles and torsion angles; correlations between esds in cell parameters are only used when they are defined by crystal symmetry. An approximate (isotropic) treatment of cell esds is used for estimating esds involving l.s. planes.

### Fractional atomic coordinates and isotropic or equivalent isotropic displacement parameters (Å^2^)

**Table:**

	*x*	*y*	*z*	*U*_iso_*/*U*_eq_	
S1	0.49912 (6)	0.15680 (2)	0.93196 (6)	0.04721 (18)	
N2	0.78333 (17)	0.11025 (6)	1.10224 (16)	0.0385 (4)	
C5	0.3217 (2)	−0.16204 (8)	0.5560 (2)	0.0395 (5)	
C15	0.5175 (2)	0.01031 (7)	0.7928 (2)	0.0385 (5)	
C12	0.4282 (2)	−0.02290 (8)	0.6785 (2)	0.0393 (5)	
C10	0.3469 (2)	−0.10671 (8)	0.5781 (2)	0.0380 (5)	
C17	0.7263 (2)	0.02401 (8)	0.9923 (2)	0.0423 (5)	
H17	0.8081	0.0101	1.0547	0.051*	
C18	0.6941 (2)	0.07646 (7)	1.0030 (2)	0.0362 (5)	
C23	0.7869 (2)	0.16369 (7)	1.0771 (2)	0.0374 (5)	
C21	0.9010 (2)	0.08767 (8)	1.2138 (2)	0.0443 (5)	
H21A	0.8654	0.0561	1.2489	0.053*	
H21B	0.9253	0.1119	1.2936	0.053*	
C28	0.6646 (2)	0.18995 (7)	0.9936 (2)	0.0381 (5)	
C16	0.6397 (2)	−0.00788 (8)	0.8909 (2)	0.0424 (5)	
H16	0.6642	−0.0428	0.8884	0.051*	
N1	0.1914 (2)	−0.11924 (7)	0.35912 (18)	0.0470 (5)	
C11	0.4296 (2)	−0.07678 (8)	0.6882 (2)	0.0394 (5)	
H11	0.4864	−0.0929	0.7692	0.047*	
C26	0.7923 (2)	0.27184 (8)	1.0287 (2)	0.0438 (5)	
C27	0.6683 (2)	0.24282 (8)	0.9705 (2)	0.0436 (5)	
H27	0.5866	0.2593	0.9152	0.052*	
C9	0.2620 (2)	−0.08213 (8)	0.4554 (2)	0.0420 (5)	
C19	0.5669 (2)	0.09442 (7)	0.9097 (2)	0.0377 (5)	
C20	0.4834 (2)	0.06242 (7)	0.8066 (2)	0.0403 (5)	
H20	0.4014	0.0762	0.7442	0.048*	
C14	0.2576 (2)	−0.02860 (8)	0.4439 (2)	0.0498 (6)	
H14	0.1999	−0.0124	0.3635	0.060*	
O1	0.7031 (2)	0.35389 (7)	0.9339 (2)	0.0900 (7)	
C13	0.3407 (2)	0.00008 (8)	0.5540 (2)	0.0482 (5)	
H13	0.3392	0.0361	0.5463	0.058*	
C24	0.9103 (2)	0.19363 (8)	1.1355 (2)	0.0444 (5)	
H24	0.9926	0.1776	1.1913	0.053*	
C6	0.2262 (2)	−0.16776 (8)	0.4193 (2)	0.0420 (5)	
C29	0.8000 (3)	0.32760 (9)	1.0036 (3)	0.0579 (6)	
H29	0.8870	0.3443	1.0448	0.069*	
C4	0.3684 (2)	−0.20599 (8)	0.6386 (2)	0.0474 (5)	
H4	0.4303	−0.2030	0.7295	0.057*	
C7	0.0799 (2)	−0.10852 (9)	0.2300 (2)	0.0535 (6)	
H7A	0.0743	−0.1375	0.1641	0.064*	
H7B	0.1074	−0.0779	0.1836	0.064*	
C25	0.9118 (2)	0.24642 (8)	1.1116 (2)	0.0474 (5)	
H25	0.9951	0.2654	1.1521	0.057*	
C1	0.1828 (2)	−0.21668 (8)	0.3631 (2)	0.0509 (6)	
H1	0.1226	−0.2204	0.2716	0.061*	
C22	1.0392 (2)	0.07482 (9)	1.1657 (2)	0.0561 (6)	
H22A	1.0222	0.0452	1.1028	0.084*	
H22B	1.1159	0.0671	1.2483	0.084*	
H22C	1.0665	0.1041	1.1156	0.084*	
C3	0.3213 (3)	−0.25401 (9)	0.5835 (3)	0.0553 (6)	
H3	0.3502	−0.2836	0.6389	0.066*	
C2	0.2315 (2)	−0.25924 (9)	0.4470 (3)	0.0560 (6)	
H2	0.2037	−0.2923	0.4116	0.067*	
C8	−0.0676 (3)	−0.09976 (12)	0.2586 (3)	0.0787 (8)	
H8A	−0.0938	−0.1292	0.3088	0.118*	
H8B	−0.1380	−0.0954	0.1690	0.118*	
H8C	−0.0654	−0.0691	0.3163	0.118*	

### Atomic displacement parameters (Å^2^)

**Table:**

	*U*^11^	*U*^22^	*U*^33^	*U*^12^	*U*^13^	*U*^23^
S1	0.0356 (3)	0.0397 (3)	0.0623 (4)	0.0083 (2)	0.0034 (3)	−0.0034 (3)
N2	0.0359 (10)	0.0368 (9)	0.0398 (9)	0.0067 (7)	0.0032 (8)	0.0026 (7)
C5	0.0376 (12)	0.0409 (11)	0.0417 (11)	0.0010 (9)	0.0125 (9)	0.0006 (9)
C15	0.0387 (12)	0.0376 (11)	0.0420 (11)	0.0009 (9)	0.0150 (9)	0.0031 (9)
C12	0.0394 (12)	0.0407 (11)	0.0399 (11)	0.0002 (9)	0.0133 (9)	0.0028 (9)
C10	0.0378 (11)	0.0404 (11)	0.0376 (11)	0.0033 (9)	0.0123 (9)	0.0023 (9)
C17	0.0382 (12)	0.0389 (11)	0.0473 (12)	0.0075 (9)	0.0052 (10)	0.0064 (9)
C18	0.0345 (11)	0.0367 (11)	0.0388 (11)	0.0034 (9)	0.0114 (9)	0.0044 (9)
C23	0.0377 (11)	0.0403 (11)	0.0346 (10)	0.0066 (9)	0.0093 (9)	−0.0006 (9)
C21	0.0448 (13)	0.0438 (12)	0.0404 (11)	0.0086 (10)	0.0023 (10)	0.0043 (9)
C28	0.0386 (12)	0.0393 (11)	0.0358 (11)	0.0042 (9)	0.0072 (9)	−0.0033 (9)
C16	0.0438 (13)	0.0337 (11)	0.0503 (12)	0.0038 (9)	0.0124 (10)	0.0026 (9)
N1	0.0502 (11)	0.0465 (10)	0.0401 (9)	−0.0018 (8)	0.0023 (8)	0.0029 (8)
C11	0.0397 (12)	0.0425 (12)	0.0362 (11)	0.0041 (9)	0.0094 (9)	0.0048 (9)
C26	0.0512 (13)	0.0419 (12)	0.0379 (11)	0.0036 (10)	0.0096 (10)	−0.0023 (9)
C27	0.0469 (13)	0.0407 (12)	0.0394 (11)	0.0081 (10)	0.0026 (10)	0.0002 (9)
C9	0.0457 (13)	0.0424 (12)	0.0380 (11)	−0.0021 (10)	0.0103 (10)	0.0034 (9)
C19	0.0353 (11)	0.0365 (11)	0.0418 (11)	0.0047 (9)	0.0104 (9)	0.0034 (9)
C20	0.0348 (11)	0.0417 (12)	0.0429 (11)	0.0041 (9)	0.0060 (9)	0.0051 (9)
C14	0.0568 (14)	0.0477 (13)	0.0408 (12)	0.0019 (11)	0.0032 (11)	0.0104 (10)
O1	0.0925 (15)	0.0504 (11)	0.1099 (16)	0.0041 (10)	−0.0101 (13)	0.0203 (11)
C13	0.0585 (15)	0.0379 (11)	0.0474 (12)	0.0016 (10)	0.0109 (11)	0.0065 (10)
C24	0.0384 (12)	0.0436 (12)	0.0476 (12)	0.0065 (10)	0.0034 (10)	−0.0025 (10)
C6	0.0371 (12)	0.0443 (12)	0.0453 (12)	−0.0021 (9)	0.0112 (9)	0.0009 (10)
C29	0.0669 (17)	0.0443 (13)	0.0594 (15)	−0.0015 (12)	0.0087 (13)	0.0006 (12)
C4	0.0461 (13)	0.0454 (13)	0.0497 (12)	0.0033 (10)	0.0095 (10)	0.0051 (10)
C7	0.0557 (15)	0.0612 (14)	0.0395 (12)	−0.0032 (11)	0.0035 (11)	0.0043 (11)
C25	0.0450 (13)	0.0477 (12)	0.0479 (12)	−0.0027 (10)	0.0076 (10)	−0.0064 (10)
C1	0.0441 (13)	0.0527 (14)	0.0539 (13)	−0.0096 (11)	0.0074 (11)	−0.0053 (11)
C22	0.0432 (13)	0.0576 (14)	0.0610 (15)	0.0145 (11)	−0.0006 (11)	−0.0043 (12)
C3	0.0539 (15)	0.0431 (13)	0.0692 (16)	−0.0002 (11)	0.0152 (13)	0.0084 (11)
C2	0.0500 (14)	0.0444 (13)	0.0740 (17)	−0.0101 (11)	0.0154 (13)	−0.0058 (12)
C8	0.0529 (17)	0.099 (2)	0.0816 (19)	0.0087 (15)	0.0108 (14)	0.0031 (17)

### Geometric parameters (Å, º)

**Table:**

S1—C28	1.759 (2)	C26—C27	1.392 (3)
S1—C19	1.7595 (19)	C26—C29	1.459 (3)
N2—C23	1.397 (2)	C27—H27	0.9300
N2—C18	1.412 (2)	C9—C14	1.381 (3)
N2—C21	1.468 (2)	C19—C20	1.381 (3)
C5—C4	1.390 (3)	C20—H20	0.9300
C5—C6	1.411 (3)	C14—C13	1.372 (3)
C5—C10	1.450 (3)	C14—H14	0.9300
C15—C16	1.387 (3)	O1—C29	1.206 (3)
C15—C20	1.392 (3)	C13—H13	0.9300
C15—C12	1.485 (3)	C24—C25	1.377 (3)
C12—C11	1.388 (3)	C24—H24	0.9300
C12—C13	1.410 (3)	C6—C1	1.392 (3)
C10—C11	1.388 (3)	C29—H29	0.9300
C10—C9	1.406 (3)	C4—C3	1.375 (3)
C17—C16	1.382 (3)	C4—H4	0.9300
C17—C18	1.392 (3)	C7—C8	1.502 (3)
C17—H17	0.9300	C7—H7A	0.9700
C18—C19	1.397 (3)	C7—H7B	0.9700
C23—C24	1.400 (3)	C25—H25	0.9300
C23—C28	1.413 (3)	C1—C2	1.371 (3)
C21—C22	1.522 (3)	C1—H1	0.9300
C21—H21A	0.9700	C22—H22A	0.9600
C21—H21B	0.9700	C22—H22B	0.9600
C28—C27	1.379 (3)	C22—H22C	0.9600
C16—H16	0.9300	C3—C2	1.386 (3)
N1—C6	1.381 (3)	C3—H3	0.9300
N1—C9	1.384 (3)	C2—H2	0.9300
N1—C7	1.449 (3)	C8—H8A	0.9600
C11—H11	0.9300	C8—H8B	0.9600
C26—C25	1.383 (3)	C8—H8C	0.9600
			
C28—S1—C19	99.26 (9)	C20—C19—S1	117.78 (14)
C23—N2—C18	121.66 (15)	C18—C19—S1	120.54 (15)
C23—N2—C21	117.98 (16)	C19—C20—C15	122.33 (18)
C18—N2—C21	118.46 (15)	C19—C20—H20	118.8
C4—C5—C6	119.35 (19)	C15—C20—H20	118.8
C4—C5—C10	134.10 (19)	C13—C14—C9	118.27 (19)
C6—C5—C10	106.54 (17)	C13—C14—H14	120.9
C16—C15—C20	115.82 (18)	C9—C14—H14	120.9
C16—C15—C12	122.90 (18)	C14—C13—C12	122.65 (19)
C20—C15—C12	121.28 (18)	C14—C13—H13	118.7
C11—C12—C13	117.99 (18)	C12—C13—H13	118.7
C11—C12—C15	122.01 (17)	C25—C24—C23	121.09 (19)
C13—C12—C15	119.99 (18)	C25—C24—H24	119.5
C11—C10—C9	119.55 (18)	C23—C24—H24	119.5
C11—C10—C5	134.33 (18)	N1—C6—C1	129.5 (2)
C9—C10—C5	106.12 (17)	N1—C6—C5	109.26 (17)
C16—C17—C18	121.50 (18)	C1—C6—C5	121.24 (19)
C16—C17—H17	119.3	O1—C29—C26	125.5 (2)
C18—C17—H17	119.3	O1—C29—H29	117.2
C17—C18—C19	116.38 (18)	C26—C29—H29	117.2
C17—C18—N2	122.63 (17)	C3—C4—C5	118.8 (2)
C19—C18—N2	120.99 (17)	C3—C4—H4	120.6
N2—C23—C24	121.61 (17)	C5—C4—H4	120.6
N2—C23—C28	121.17 (18)	N1—C7—C8	113.25 (19)
C24—C23—C28	117.21 (18)	N1—C7—H7A	108.9
N2—C21—C22	115.14 (17)	C8—C7—H7A	108.9
N2—C21—H21A	108.5	N1—C7—H7B	108.9
C22—C21—H21A	108.5	C8—C7—H7B	108.9
N2—C21—H21B	108.5	H7A—C7—H7B	107.7
C22—C21—H21B	108.5	C24—C25—C26	121.5 (2)
H21A—C21—H21B	107.5	C24—C25—H25	119.3
C27—C28—C23	120.91 (19)	C26—C25—H25	119.3
C27—C28—S1	118.57 (15)	C2—C1—C6	117.9 (2)
C23—C28—S1	120.23 (15)	C2—C1—H1	121.1
C17—C16—C15	122.44 (18)	C6—C1—H1	121.1
C17—C16—H16	118.8	C21—C22—H22A	109.5
C15—C16—H16	118.8	C21—C22—H22B	109.5
C6—N1—C9	108.37 (16)	H22A—C22—H22B	109.5
C6—N1—C7	125.54 (18)	C21—C22—H22C	109.5
C9—N1—C7	125.18 (18)	H22A—C22—H22C	109.5
C12—C11—C10	120.52 (18)	H22B—C22—H22C	109.5
C12—C11—H11	119.7	C4—C3—C2	121.3 (2)
C10—C11—H11	119.7	C4—C3—H3	119.4
C25—C26—C27	118.33 (19)	C2—C3—H3	119.4
C25—C26—C29	119.6 (2)	C1—C2—C3	121.4 (2)
C27—C26—C29	122.1 (2)	C1—C2—H2	119.3
C28—C27—C26	121.00 (19)	C3—C2—H2	119.3
C28—C27—H27	119.5	C7—C8—H8A	109.5
C26—C27—H27	119.5	C7—C8—H8B	109.5
C14—C9—N1	129.31 (18)	H8A—C8—H8B	109.5
C14—C9—C10	121.02 (19)	C7—C8—H8C	109.5
N1—C9—C10	109.67 (17)	H8A—C8—H8C	109.5
C20—C19—C18	121.40 (18)	H8B—C8—H8C	109.5
			
C16—C15—C12—C11	21.4 (3)	C5—C10—C9—C14	178.0 (2)
C20—C15—C12—C11	−159.25 (19)	C11—C10—C9—N1	178.85 (18)
C16—C15—C12—C13	−157.3 (2)	C5—C10—C9—N1	−2.0 (2)
C20—C15—C12—C13	22.1 (3)	C17—C18—C19—C20	−4.0 (3)
C4—C5—C10—C11	1.8 (4)	N2—C18—C19—C20	176.20 (18)
C6—C5—C10—C11	−179.4 (2)	C17—C18—C19—S1	169.74 (15)
C4—C5—C10—C9	−177.1 (2)	N2—C18—C19—S1	−10.1 (3)
C6—C5—C10—C9	1.7 (2)	C28—S1—C19—C20	−151.56 (16)
C16—C17—C18—C19	2.3 (3)	C28—S1—C19—C18	34.50 (18)
C16—C17—C18—N2	−177.84 (18)	C18—C19—C20—C15	2.3 (3)
C23—N2—C18—C17	153.75 (19)	S1—C19—C20—C15	−171.57 (16)
C21—N2—C18—C17	−10.2 (3)	C16—C15—C20—C19	1.1 (3)
C23—N2—C18—C19	−26.4 (3)	C12—C15—C20—C19	−178.31 (19)
C21—N2—C18—C19	169.58 (18)	N1—C9—C14—C13	−178.6 (2)
C18—N2—C23—C24	−152.34 (19)	C10—C9—C14—C13	1.3 (3)
C21—N2—C23—C24	11.7 (3)	C9—C14—C13—C12	−0.9 (3)
C18—N2—C23—C28	28.8 (3)	C11—C12—C13—C14	0.2 (3)
C21—N2—C23—C28	−167.15 (17)	C15—C12—C13—C14	178.9 (2)
C23—N2—C21—C22	−83.2 (2)	N2—C23—C24—C25	−179.10 (19)
C18—N2—C21—C22	81.4 (2)	C28—C23—C24—C25	−0.2 (3)
N2—C23—C28—C27	179.27 (18)	C9—N1—C6—C1	−179.4 (2)
C24—C23—C28—C27	0.4 (3)	C7—N1—C6—C1	11.1 (4)
N2—C23—C28—S1	5.6 (3)	C9—N1—C6—C5	−0.6 (2)
C24—C23—C28—S1	−173.33 (15)	C7—N1—C6—C5	−170.09 (19)
C19—S1—C28—C27	153.97 (16)	C4—C5—C6—N1	178.28 (19)
C19—S1—C28—C23	−32.19 (17)	C10—C5—C6—N1	−0.7 (2)
C18—C17—C16—C15	1.1 (3)	C4—C5—C6—C1	−2.8 (3)
C20—C15—C16—C17	−2.8 (3)	C10—C5—C6—C1	178.24 (19)
C12—C15—C16—C17	176.64 (19)	C25—C26—C29—O1	−179.7 (2)
C13—C12—C11—C10	0.0 (3)	C27—C26—C29—O1	0.9 (4)
C15—C12—C11—C10	−178.71 (18)	C6—C5—C4—C3	0.8 (3)
C9—C10—C11—C12	0.4 (3)	C10—C5—C4—C3	179.5 (2)
C5—C10—C11—C12	−178.4 (2)	C6—N1—C7—C8	87.7 (3)
C23—C28—C27—C26	0.0 (3)	C9—N1—C7—C8	−80.1 (3)
S1—C28—C27—C26	173.83 (16)	C23—C24—C25—C26	−0.4 (3)
C25—C26—C27—C28	−0.6 (3)	C27—C26—C25—C24	0.8 (3)
C29—C26—C27—C28	178.8 (2)	C29—C26—C25—C24	−178.6 (2)
C6—N1—C9—C14	−178.4 (2)	N1—C6—C1—C2	−178.9 (2)
C7—N1—C9—C14	−8.8 (4)	C5—C6—C1—C2	2.4 (3)
C6—N1—C9—C10	1.7 (2)	C5—C4—C3—C2	1.4 (3)
C7—N1—C9—C10	171.23 (19)	C6—C1—C2—C3	−0.1 (3)
C11—C10—C9—C14	−1.1 (3)	C4—C3—C2—C1	−1.9 (4)

### Hydrogen-bond geometry (Å, º)

Cg3 and Cg4 are the centroids of the C1–C6 and C9–C14 rings, respectively.

**Table:**

*D*—H···*A*	*D*—H	H···*A*	*D*···*A*	*D*—H···*A*
C21—H21*A*···*Cg*4^i^	0.97	2.95	3.596 (2)	125
C25—H25···*Cg*3^ii^	0.93	2.96	3.558 (2)	123

Symmetry codes: (i) −*x*+1, −*y*, −*z*+2; (ii) −*x*+3/2, *y*+1/2, −*z*+3/2.
